# Ethnic Differences in MicroRNA-375 Expression Level and DNA Methylation Status in Type 2 Diabetes of Han and Kazak Populations

**DOI:** 10.1155/2014/761938

**Published:** 2014-03-11

**Authors:** Xiangyun Chang, Siyuan Li, Jun Li, Liang Yin, Ting Zhou, Chen Zhang, Xuan Chen, Kan Sun

**Affiliations:** ^1^Departments of Endocrinology and Metabolism, First Affiliated Hospital, Shihezi University School of Medicine, Shihezi, Xinjiang 832002, China; ^2^Tongji Hospital, Tongji Medical College, Huazhong University of Science and Technology, Wuhan 430030, China; ^3^Shihezi University School of Medicine, Shihezi, Xinjiang 832002, China; ^4^The Central Laboratory, First Affiliated Hospital, Shihezi University School of Medicine, Shihezi, Xinjiang 832002, China

## Abstract

Han population is six times as likely as Kazak population to present with type 2 diabetes mellitus (T2DM) in China. We hypothesize that differential expression and CpG methylation of miR-375 may be an ethnic-related factor that influences the incidence of T2DM. The expression level of miR-375 was examined using real-time PCR on Kazak and Han T2DM plasma samples. Furthermore, the methylation levels of CpG sites of miR-375 promoter were determined by MassARRAY Spectrometry in these samples. The relative expression levels of plasma miR-375 in Kazak T2DM samples are 1, and the relative expression levels of plasma miR-375 in Han T2DM samples are 3. The mean level of miR-375 methylation, calculated from the methylation levels of the CpG sites, was 8.47% for the Kazak T2DM group and 10.38% for the Han T2DM group. Further, five CpG units showed a statistically significant difference between Kazak and Han T2DM samples, and, among them, four were hypomethylated and only one CpG unit showed hypermethylation in Kazak T2DM samples. These findings indicate that the expression levels of plasma miR-375 and its CpG methylation in the promoter region are ethnically different, which may contribute to the different incidence of diabetes observed in Kazak and Han populations.

## 1. Introduction

Type 2 diabetes mellitus (T2DM) is commonly associated with obesity and results from defects in insulin secretion and/or diminished sensitivity of target tissues to insulin action. MicroRNAs (miRNAs) are endogenous about 22 nucleotides noncoding RNAs. They participate in posttranscriptional regulation of gene expression, therefore, to directly control the expression of a large portion of the human genome [1]. Studies showed that miRNAs are involved in the regulation of major cellular activities, such as metabolism, differentiation, proliferation, and apoptosis [2, 3].

miRNAs are also important regulators of specialized *β*-cell functions [4–7]. Indeed, expression of appropriate levels of miR-375, miR-9, and miR-124a are required for insulin biosynthesis and for optimal release of insulin in response to secretagogues [4–7]. The dysregulated expressions of several miRNAs have been reported in the development of diabetes. In particular, miR-375 is selectively expressed in pancreatic islets [4, 5] and regulates insulin secretion and islet *β*-cell proliferation to participate in normal glucose homeostasis. Its abnormal expression may be caused by pancreatic *β*-cell dysfunction. The plasma/serum level of miRNAs can be present in a remarkably stable form and the expression level of serum miRNAs is reproducible and consistent among individuals [8]. We hypothesised that plasma miR-375 concentration contributed to potential biomarkers in patients with T2DM. However, to date, there has not been any report on the role of circulating miR-375 in plasma of patients with T2DM and the exact mechanisms for its aberrant expression remain unclear.

In addition to classical genetic abnormalities in the pathogenesis of T2DM, epigenetic modifications have emerged as a central driving force for the development of T2DM. Epigenetic modifications, especially DNA hypermethylation, are believed to play an important role in the regulation of essential protective genes for T2DM. Found in the tumor area, epigenetic regulations of miRNA genes within or near CpG islands in their promoter regions can result in aberrant expressions of a number of miRNAs.* miR-375* gene is located in the intergenic region and it has an independent promoter containing CpG islands, which provides a structural basis for regulation of its expression by methylation. Thus, we speculate that the aberrant methylation of human* miRNA-375* gene promoter may lead to their abnormal expression, thereby causing *β*-cell dysfunction to ultimately participate in the pathophysiological process of T2DM.

In China, Kazak population is significantly overweight and has insulin resistance, hypertension, smoking, and other risk factors for T2DM, but its T2DM prevalence rate is much lower than the Han population in the same region [9]. Unique genetic background of Kazak population may protect them from T2DM. To identify the possible genetic difference of Kazak population from Han population, we hypothesized that differential expression and CpG methylation of miR-375 may be an ethnic-related factor that influences the incidence and severity of T2DM in this two ethnic groups.

## 2. Materials and Methods 

### 2.1. Patients

This study was prospectively performed and approved by the Institutional Ethics Committees of the First Affiliated Hospital of Shihezi University School of Medicine in China and conducted in accordance with the ethical guidelines of the Declaration of Helsinki. One hundred patients with T2DM from Kazak population and another 100 T2DM from Han population were recruited from the Departments of Endocrinology and Metabolism of First Affiliated Hospital of Shihezi University School of Medicine in China from 2010 to 2011. Written informed consents were obtained from all patients before they entered the study. Diagnosis of type 2 diabetes was based on the World Health Organization criteria: fasting glucose ≥ 7 mmol/L (126 mg/dL) or the 2-hour oral glucose tolerance test glucose level ≥ 11.1 mmol/L (200 mg/dL) or clinical diagnosis of the disease. All subjects were recruited when they were hospitalized for treatment of poor glycemic control and any patients suspected of infectious disease or autoimmune disease were excluded from current study.

### 2.2. Nucleic Acid Isolation

RNAs were isolated from plasma samples of Kazak and Han T2DM patients using the miRNeasy Serum/Plasma Kit (Qiagen, Germany) and genomic DNA was isolated from blood cells using the DNeasy Blood and Tissue Kit (Qiagen, Germany) according to manufacturer's instruction. The nucleic acid samples were quantified by measuring their absorption at 260 nm.

### 2.3. Reverse Transcription and Quantitative PCR

Real-time reverse transcription PCR (RT-PCR) was performed using an ABI Prism 7500 Fast Real-time PCR System with Taqman Universal PCR Master Mix, Taqman Reverse Transcription kit, Taqman MicroRNA Assays, and Human Panel Early Access kit from Applied Biosystems (Foster City, CA) according to the manufacturer's instructions. Expression levels of miRNA genes were determined by normalizing the amount of the target message to that of the control microRNA-16 transcript.

### 2.4. Sequenom Methylation Analysis

To quantify methylation levels of the miR-375 CpG islands in the clinical samples, the high-throughput MassARRAY platform (Sequenom, San Diego, USA) was carried out as described previously [10]. Briefly, primers for the miR-375 CpG island were used to amply bisulfite treated DNA and then the PCR products were spotted on a 384-pad SpectroCHIP (Sequenom, San Diego, USA), followed by spectral acquisition on a MassARRAY Analyzer. Methylation data of individual units (one to three CpG sites per unit) were generated by the EpiTyper v1.0.5 software (Sequenom, San Diego, USA).

### 2.5. Statistical Methods

The associations between categorical variables were assessed using the *χ* ^2^ test. Significance level was set as *P* value < 0.05. In the analysis of the Chinese Kazak T2DM samples, whether the differences in miR-375 gene methylation were differently depending on clinical parameters was determined by the Mann-Whitney test. The distances between CpG methylation sites to transcription start sites were calculated by using the RMySQL package and the SQL database version of the UCSC genome browser (http://genome.ucsc.edu/cgi-bin/hgGateway). Two-dimensional clustering was determined by the heatmap.2 function in the gregmisc package. Classical multidimensional scaling had been performed by using the cmdscale function, and visualization was done through the scatter plot3d function in the same package. Power calculations were done using the software of Power and Sample Size Calculation 3.0 (USA). We calculated the Spearman correlation between DNA methylation levels and plasma levels of miR-375. Tests for statistical significance had been used with standard function in* R* statistical environment.

## 3. Results

### 3.1. Lower Expression of miR-375 in Kazak T2DM Compared with Han T2DM

**Figure 1:**
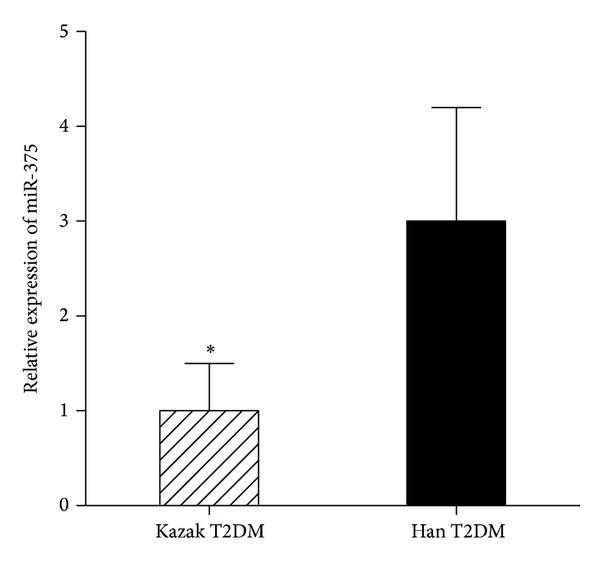
The levels of* miR-375* expression in Kazak and Han T2DM samples. The plasma mRNA levels of* miR-375 *in 100 Kazak and 100 Han T2DM samples were determined by quantitative real-time PCR as described in Materials and Methods. The relative expression values were defined as the expression ratio of* miR-375* to* miR-16*. The values represented average expression levels with standard deviation (SD) of three independent experiments and the significant level was determined by Student's* t*-tests between the indicated groups (*P < 0.05).

To find out if there is difference in miR-375 expression levels between Kazak and Han populations, the expression levels of miR-375 in Chinese Kazak T2DM samples and corresponding Han T2DM samples were determined by quantitative real-time PCRs.  Figure 1 showed that the relative expression levels of plasma miR-375 in Kazak T2DM samples are 1, and the relative expression levels of plasma miR-375 in Han T2DM samples are 3 (*P* < 0.05).

### 3.2. CpG Methylation of miR-375 in Kazak and Han Populations

**Figure 2:**
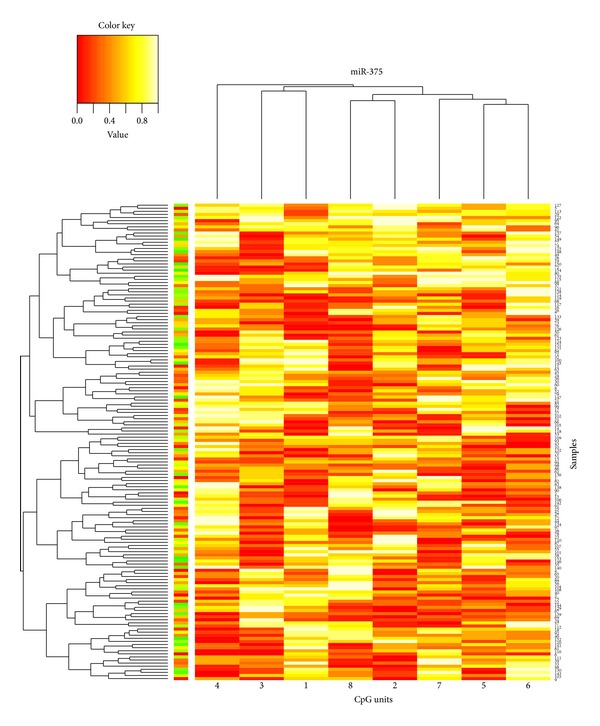
Hierarchical clustering of miR-375 methylation profiles in Kazak and Han T2DM samples. MassARRAY analysis quantified DNA methylation status of miR-375 gene promoter region in Kazak and Han T2DM samples. Each row represented a sample and each column represented a CpG unit, which was a single CpG site or a combination of CpG sites. Color coding reflected the degree of methylation with yellow being 100% red being 0%, and gray being no data.

In order to know whether CpG methylation of miR-375 occurs in Kazak and Han populations, methylation patterns of miR-375 in 100 samples from each population were quantitatively analyzed using MassARRAY Spectrometry. Hierarchical clustering showed substantial differences in the quantitative methylation profiling of Kazak T2DM cases compared with Han T2DM cases (Figure 2).

**Figure 3:**
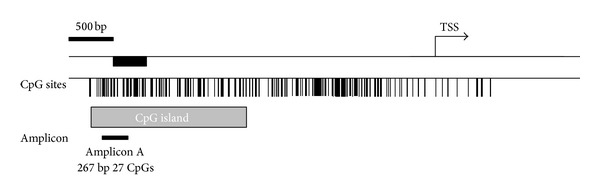
Illustrative map of* miR-375* promoter region. Vertical lines depict the CpG dinucleotides. The arrow indicates the transcriptional start site. Gray filled box is CpG island and black filled bars are MassARRAY studied amplicon regions. Amplicon characteristics are shown beneath the black bars and bp indicates base pair.

**Figure 4:**
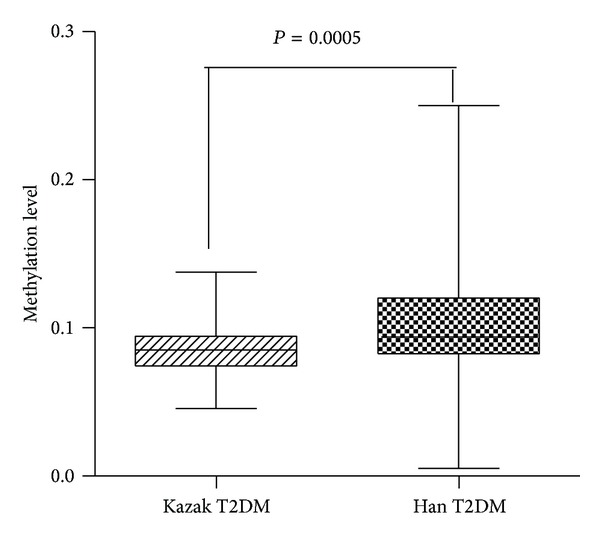
The DNA methylation levels of* miR-375* promoter in the samples from Kazak and Han patients with T2DM. The mean methylation levels of the 17 CpG residues in 100 samples from each population were determined by MassARRAY and the data are presented as mean ± standard deviation (SD).

The promoter region of miR-375 was mapped by UCSC Genome Browser's CpG island annotations and miRbase release 13.0. As illustrated in Figure 3, methylations were found in the region from −990 bp to −1,258 bp relative to the transcription start site of* miR-375 *in Chinese Kazak T2DM samples. The mean methylation level of the 17 CpG residues was 8.47% for the Kazak T2DM patients and 10.38% for the Han patients, which was significantly different with *P* = 0.0005 (Figure 4). The Kazak T2DM samples showed various levels of* miR-375* promoter methylation, ranging from 3.0% to 18.5%. In addition, we used the methylation level to characterize relationships among the T2DM samples and to explore potential associations with their clinical features. Our study found that none of the clinical parameters (BMI, WHR, SBP, FBG, LDL-C, TC, TG, and lower HDL-cholesterol) had statistically significant differences on the methylation status of the miR-375 promoter (data is not shown).

**Figure 5:**
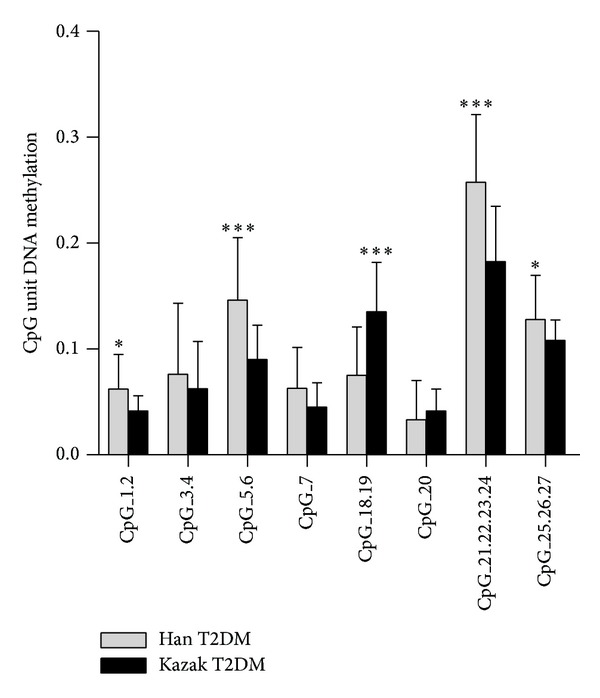
Comparison of specific* miR-375* CpG methylation in Kazak and Han T2DM samples. The average methylation levels of specific CpG units of amplicon from 100 samples in each T2DM groups were determined by MassARRAY EpiTyper. ***P < 0.001 and *P < 0.05. The error bars represented standard error.

We noted heterogeneity among individual CpG units, so eight CpG units of the total 17 analyzed CpG units were further analyzed and they span 267 bp on the promoter region of* miR-375*. Among the eight CpG units, five showed a statistically significant difference between Kazak and Han T2DM patients. And four of the five CpGs were hypomethylated (CpG 1.2, CpG 3.4, CpG 5.6, and CpG 21.22.23.34), while only one CpG unit (CpG 18.19) showed hypermethylation in Kazak T2DM patients (Figure 5).

### 3.3. Correlation between Promoter Methylation and the Expression of Plasma miR-375

**Figure 6:**
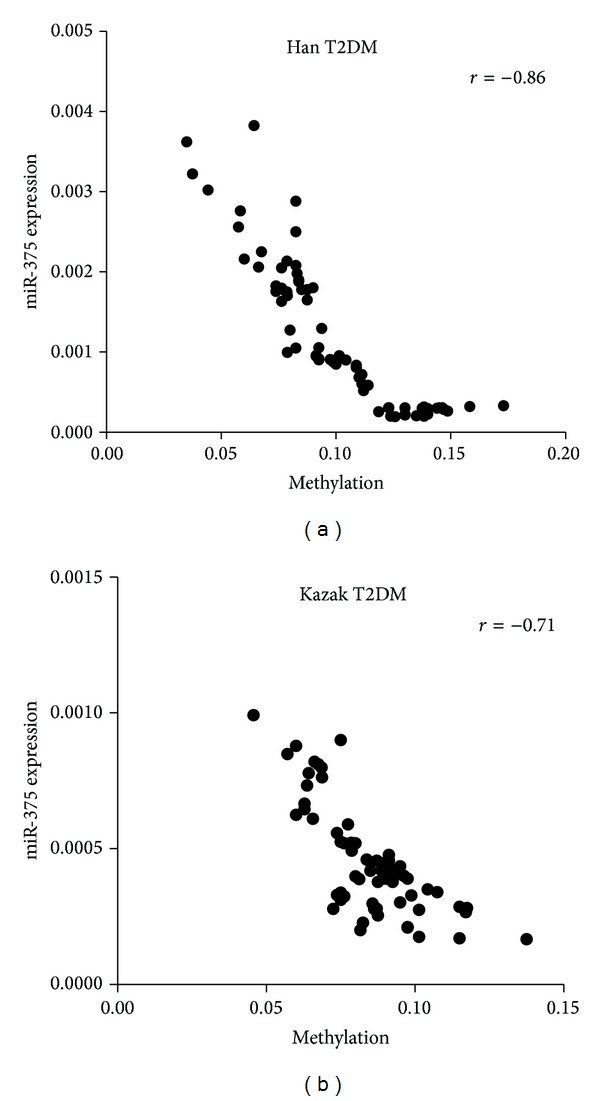
The correlation analysis between CpG methylation levels of miR-375 promoter and plasma levels of miR-375. An inverse correlation for clear trend towards a negative correlation between DNA methylation levels and plasma levels of miR-375 is shown for each of the two sample sets: (a) Kazak T2DM and (b) Han T2DM. Spearman correlation coefficients are reported. *P* values for both samples are significant (*P* < 0.001).

To further explore the role of methylation, we analyzed the correlation analysis between CpG methylation levels of miR-375 promoter and plasma levels of miR375. In Kazak and Han T2DM patients, we found an inverse correlation for clear trend towards a negative correlation between DNA methylation levels and plasma levels of miR-375 (Figure 6(a), Pearson *r* = −0.71; Figure 6(b), Pearson *r* = −0.86). These suggest the role of methylation in the regulation of expression of miR-375.

## 4. Discussion

Han population is six times as likely as Kazak population to present with T2DM in China [9]. The reason for this ethical difference is not well understood. Detailed molecular epidemiological studies that consider behavioral and environmental risk factors, inherited susceptibility, and acquired molecular alterations are needed to elucidate the etiology of this heterogeneous disease in both Han and Kazak populations.

Some recent studies show that DNA methylation contributes to downregulation of miR-375 during tumorigenesis [6, 7, 11]. However, it is not clear whether miR-375 promoter methylation plays a role in T2DM. We hypothesized that differential expression and CpG methylation of miR-375 may be an ethnic-related factor that influences the incidence and severity of T2DM.

In this study, we found that the plasma level of miR-375 was significantly lower in Kazak T2DM samples compared to Han T2DM samples (Figure 1). It has been shown previously that overexpression of miR-375 downregulates the expression of myotrophin, a known regulator of catecholamine release [12]. Together, these observations may suggest that miR-375 is involved in the pathogenesis of T2DM. Since the plasma/serum level of miRNAs can be present in a remarkably stable form and the expression level of serum miRNAs is reproducible and consistent among individuals, earlier study demonstrated the presence of circulating miRNAs and their potential use as novel biomarkers of diseases [8]. Our data suggested that the plasma level of miR-375 in Chinese Kazak T2DM individuals might be able to distinguish them from Han T2DM, and, therefore, miR-375 could be used as a novel ethnic-related biomarker of T2DM in Chinese T2DM population.

Epigenetic modification of DNA, such as methylation, is thought to play a key role in T2DM progression and miRNAs are epigenetically regulated by DNA methylation [7]. To date, several studies have revealed that the expression of miR-375 is epigenetically regulated in hepatocellular, gastric, and breast cancers [6, 13]. Our previous study demonstrated that the miR-375 promoter is hypomethylated, in Kazak patients with T2DM, which may regulate the expression of miR-375 and contribute to the pathogenesis of T2DM [14], but no studies have directly linked miR-375 promoter methylation to T2DM and demonstrate its ethnic disparity in the entire group of studied samples. In the present study, using the high-throughput robust T-cleavage assay on MALDI-TOF MS, we showed hypomethylation patterns in Chinese Kazak T2DM samples compared with Han T2DM samples. This ethnic difference in the frequency of methylation existed in the entire group of samples (*P* = 0.0005, Figure 4). The study examined correlations between promoter methylation and the expression of plasma miR-375. We found for miR-375 that decreased DNA methylation was correlated with increased levels of transcription of miR-375 in Kazak and Han T2DM samples (Figures 6(a) and 6(b)). The association between DNA methylation and gene expression was much stronger among T2DM samples than among Kazak T2DM samples. Thus, it implied that studying the epigenetic makeup of different ethnic groups may help to elucidate pathogenic mechanisms of T2DM.

We further evaluated the methylation status of the CpG units to test the possibility to use them to predict the progression of Kazak T2DM. Our results showed statistically significant differences in the frequency of methylation at individual CpG units between Kazak and Han T2DM samples. And more than half of the studied individual CpG units were hypomethylated in Kazak T2DM patients, CpG 1.2, CpG 3.4, CpG 5.6, and CpG 21.22.23.34, as compared with Hans (*P* < 0.05). Despite the small sample size, this is the first study, to the best of our knowledge, to report a possibility that the methylation frequency at individual CpG units may serve as novel diagnostic ethnic markers used to distinguish Kazak T2DM patients from Hans.

In summary, we demonstrated, in this study, the presence of ethnic differences in the expressions of miR-375 and CpG methylation of miR-375 promoter regions, and this difference may contribute to the different patterns of T2DM frequency observed in Kazak and Han populations. Meanwhile, miR-375 methylation patterns could be added to the known risk factors in predicting the progression of Kazak T2DM.
